# Acute Hepatitis E Virus Infection in Two Geographical Regions of Nigeria

**DOI:** 10.1155/2017/4067108

**Published:** 2017-12-13

**Authors:** I. M. Ifeorah, T. O. C. Faleye, A. S. Bakarey, M. O. Adewumi, A. Akere, E. C. Omoruyi, A. O. Ogunwale, J. A. Adeniji

**Affiliations:** ^1^Department of Medical Laboratory Sciences, Faculty of Health Science and Technology, College of Medicine, University of Nigeria, Nsukka, Nigeria; ^2^Department of Microbiology, Faculty of Science, Ekiti State University, Ado Ekiti, Nigeria; ^3^Department of Virology, College of Medicine, University of Ibadan, Ibadan, Nigeria; ^4^Institute for Advanced Medical Research & Training, College of Medicine, University of Ibadan, Ibadan, Nigeria; ^5^Department of Medicine, College of Medicine, University of Ibadan, Ibadan, Nigeria; ^6^Institute of Child Health, College of Medicine, University of Ibadan, Ibadan, Nigeria; ^7^Oyo State College of Agriculture and Technology, Igboora, Nigeria

## Abstract

Hepatitis E virus (HEV) remains a major public health concern in resource limited regions of the world. Yet data reporting is suboptimal and surveillance system is inadequate. In Nigeria, there is dearth of information on prevalence of acute HEV infection. This study was therefore designed to describe acute HEV infection among antenatal clinic attendees and community dwellers from two geographical regions in Nigeria. Seven hundred and fifty plasma samples were tested for HEV IgM by Enzyme Linked Immunosorbent Assay (ELISA) technique. The tested samples were randomly selected from a pool of 1,115 blood specimens previously collected for viral hepatitis studies among selected populations (pregnant women, 272; Oyo community dwellers, 438; Anambra community dwellers, 405) between September 2012 and August 2013. One (0.4%) pregnant woman in her 3rd trimester had detectable HEV IgM, while community dwellers from the two study locations had zero prevalence rates of HEV IgM. Detection of HEV IgM in a pregnant woman, especially in her 3rd trimester, is of clinical and epidemiological significance. The need therefore exists for establishment of a robust HEV surveillance system in Nigeria and especially amidst the pregnant population in a bid to improve maternal and child health.

## 1. Introduction

Hepatitis E virus (HEV) is a small, nonenveloped spherical particle of about 32–34 nm diameter and has a single stranded, positive sense RNA genome of approximately 7.5 kb that is surrounded by an icosahedral capsid [1]. It is the only member of the genus* Hepevirus* and has four genotypes capable of causing human infection [2, 3]. Hepatitis E virus (HEV) infection is a major public health concern especially in sub-Saharan Africa among other developing countries [4, 5]. It is one of the most common causes of acute hepatitis and jaundice in the world and has affected about one-third of the world's population [6]. Annually, about 20 million new cases and 33 million acute cases of HEV infection occur globally and HEV-related hepatic failure is responsible for approximately 56,600 deaths per year [7, 8].

While it is established that HEV transmission occurs predominantly via the fecal-oral route [9, 10], parenteral, person-to-person, perinatal mode of transmission and eating undercooked wild boar, deer, and pork have also been suggested [11–14]. In developing countries, infection occurs both sporadically and as an epidemic, affecting a large proportion of the population [15].

In human population, prevalence of anti-HEV varies from one population to another, with significant increase noted among pregnant women and children <2 years old [4]. HEV infection is usually asymptomatic and self-limiting [16]; however, in some cases, acute infection may develop to fulminant hepatitis with high mortality especially among pregnant women in their third trimester [17], even as chronic forms of HEV have been documented among different populations [18–20]. Diagnosis of HEV infection is based on detection of anti-HEV IgM, anti-HEV IgG, and HEV RNA. Specifically, the presence of anti-HEV IgM is a marker of acute HEV infection [21].

Despite the significance of HEV in public health, especially in resource limited regions of the world, data reporting remained suboptimal, consequent of inadequate surveillance system [22]. In Nigeria, there exists dearth of information on prevalence and circulation of HEV infection in the population. In a bid to bridge this knowledge gap, we attempted, in this study, to describe acute HEV infection among selected population groups in two geographical regions in Nigeria.

## 2. Methodology

**Figure 1:**
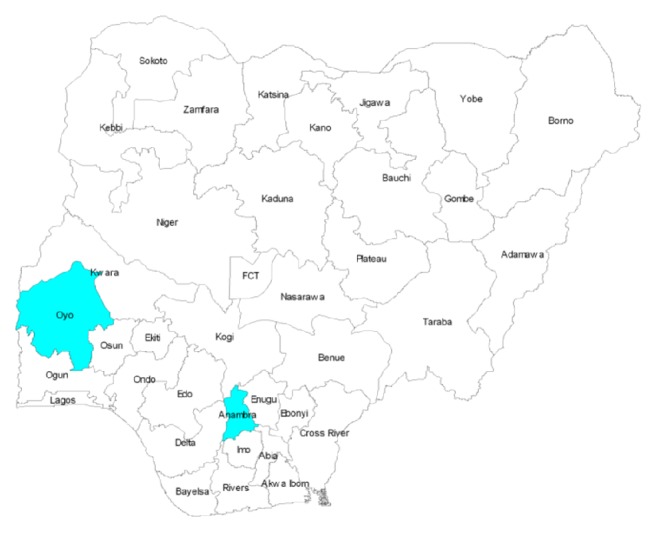
Map of Nigeria showing the study communities.

Seven hundred and fifty (750) plasma samples were randomly selected from a pool of 1,115 samples previously collected for viral hepatitis studies [23–27]. The 1,115 plasma samples were collected from consenting community dwellers and antenatal clinic attendees who participated in our previous studies conducted between September 2012 and August 2013. Specifically, the study populations include (1) pregnant women (*n* = 272) attending antenatal clinic in two different hospitals in Ibadan, Oyo state, Southwest, Nigeria, (2) community dwellers (*n* = 438) residing in and around Ibadan, Oyo state, Southwest, Nigeria, and (3) community dwellers (*n* = 405) from three selected communities in Anambra state, Southeast Nigeria (Figure 1). These samples were stored at −86°C at the Department of Virology, College of Medicine, University of Ibadan, Nigeria. A total of 250 plasma samples per population were randomly selected to make the 750 samples analyzed in this study.

All samples were screened for HEV IgM using Enzyme Linked Immunosorbent Assay (ELISA) kits (Wantai Biological immunoassay, Beijing, China) with documented sensitivity and specificity of 97.10% and 98.40%, respectively. Assays were performed according to manufacturer's instructions. Optical density was read using the Emax endpoint ELISA microplate reader (Molecular Devices, California, USA) and the results interpreted accordingly. Demographic and other relevant information were obtained from the participants using a well-structured questionnaire. Ethical approval for the study was granted by Ul/UCH Ethics committee (UI/UCH/1 1/0058), Oyo State Ministry of Health (A03/479/349), and Anambra State Ministry of Health (MH/PHD/MISC/1).

## 3. Results

In the pregnant women cohort (age range: 17–43 years; median age: 29 years), one (0.4%) participant had detectable HEV IgM. Majority (50%) of the pregnant women, including the only positive participant, are within age group 31–40 years. Further analysis shows 2%, 44.8%, and 3.2% for age groups 15–20, 21–30, and 41–45 years, respectively (Table 1). Forty-four (17.6%), 74 (29.6%), and 132 (52.8%) of the pregnant women were in their 1st, 2nd, and 3rd trimester of gestation, respectively. It is noteworthy that the participant with detectable HEV IgM was in her 33rd week (3rd trimester) of gestation (Table 2).

Overall, the Ibadan community dwellers (age range: 1.5–87 years; median age: 40 years) and the Anambra communities' residents (age range: 15–70 years; median age: 28.4 years) had zero prevalence rate for HEV IgM. Altogether, 70.8% and 29.2% of the Ibadan community dwellers were females and males, respectively. Further, the highest (25.6%) and lowest (16.6%) frequencies were recorded among age groups 21–30 and ≤20 years, respectively. For the Anambra residents, 54.8% were females, while 45.2% were males. Also, the highest (29.6%) frequency was recorded in age group 21–30 years, while age groups ≤20 had the lowest (0.05%) (Table 1).

## 4. Discussion

In this study, we report HEV IgM rate of 0.4% in the pregnant women cohort and zero prevalence rate among community dwellers in two different geographical regions of Nigeria. Our finding is in congruence with the rates of 0.9% reported in a study among different populations including apparently healthy individuals in Plateau state [28] but differs from 1.7% reported among ARV naïve and experienced HIV patients in Ibadan [29]. Though varied rates of HEV IgM have been reported in other endemic regions of the world among different populations [30–32]. The reasons for this discrepancy may range from variation in populations studied and test kits used among others.

Furthermore, wide variations in reported seroprevalence of HEV antibodies in Africa during nonepidemic periods of acute and symptomatic hepatitis have been documented [33]. Additionally, studies have indicated that commercially available ELISA kits for anti-HEV antibodies differ dramatically in their sensitivity and specificity [34–36]. In our study, we used an ELISA kit that has been confirmed to have the highest diagnostic sensitivity and specificity when compared to many others by several studies [35, 37]. Specifically, Vollerman et al. [38] in a comparative study to monitor HEV antibody seroconversion in asymptomatically infected blood donors using 9 different commercially available ELISA kits reported the best performance with the kit used in this study. Particularly, considerable variation for detection period of IgM antibodies was noted. Therefore, this implies that the prevalence rates of anti-HEV IgM recorded in this study are likely true representative of the study population as at the time of testing.

Results of this study might imply that the studied communities have good supply of clean water, as drinking of fecal contaminated water has been implicated as one of the major routes of HEV transmission [39]. Further, considering that HEV is known to cause acute infection [4], a study among asymptomatic persons may not capture acutely infected individuals who might be too sick to participate in such studies. However, this report does not underestimate the burden of acute HEV infection, especially, in resource limited countries as recent reports have confirmed outbreaks with dire consequences in different parts of sub-Saharan Africa including the Northern part of Nigeria [40, 41]. This therefore calls for the establishment of a robust surveillance system with focus on HEV that currently seems to be neglected.

The rate of 0.4% found among the pregnant women is in agreement with the rates of 0.5%, 0.6%, and 0.67% reported among pregnant women in different regions of the world including Africa [42–44]. Our finding, though higher than 0% rate reported in a similar cohort in France [45], is lower than rates ranging from 2.6% to 33% recorded by several authors in a similar cohort in different regions of the world [46–50]. Variation in the performance of different ELISA kits employed in these studies may account for the difference in rates reported.

The fact that the only pregnant woman with HEV IgM in this study was in her 33rd week of gestation signifies a recent exposure to HEV and the medical significance of the case. The reason being that acute HEV in pregnancy can progress to fulminant hepatitis with a high mortality rate, especially, if it occurs in the 3rd trimester [17, 51]. Further, reports have shown that HEV infection during pregnancy can lead to maternal mortality rate of 15% to 25%, especially, with genotype 1, which together with genotype 2 are prevalent in the developing countries [52].

Acute HEV infection during pregnancy is associated with dire consequences for the unborn, including high rate of miscarriage, stillbirth, neonatal death, high risk of vertical transmission, and high risk of preterm delivery with poor neonatal survival rates [51, 53, 54]. It was reported that HEV infection might be responsible for 2,400 to 3,000 still births each year in developing countries, with many additional fetal deaths linked to antenatal maternal deaths [55]. It is worth mentioning that we do not have information on the outcome of this singular woman's pregnancy. Hence, we could not document the impact of the HEV infection in the 3rd trimester of pregnancy on either the mother or child.

Recent outbreaks of HEV infection have been documented among Nigerian refugees in Chad and in the Northern part of Nigeria with a significant number of pregnant women being affected [40, 41, 56]. With all the health challenges posed by acute HEV infection (especially among pregnant women) it is still grossly underreported in developing countries including Nigeria. For example, there are no laid down policies on HEV management in the country. The pregnant woman with detectable HEV IgM has documented report of fever and jaundice within the preceding 2 weeks prior sampling. Thus, it is probable that the symptoms were as a result of HEV infection, since the woman tested negative to other viral infections with the possibility of showing similar symptoms [23]. Laboratory screening for acute HEV infection is rarely done in Nigeria and more scarcely among pregnant women even when presenting with symptoms suggestive of hepatitis infection (especially when there is no outbreak). Routinely, HBV is the only hepatitis virus pregnant women are screened for in Nigeria. Therefore, it is safe to assume that screening for HEV IgM was not carried out for this woman at the antenatal clinic. Thus, she obviously might not have been managed for HEV infection. This brings to light how acute HEV infections are possibly missed despite its clinical and epidemiological implications for the subject and the population, respectively.

## 5. Conclusion

We documented acute HEV infection in a pregnant woman in Ibadan, Oyo State, Southwest Nigeria, and reported zero prevalence rates of acute HEV infection among community dwellers in two different locations in the country. Consequently, we recommend more extensive studies on HEV infection in the population in a bid to define the dynamics of this neglected viral infection. In addition, we recommend the introduction of HEV IgM screening for pregnant women in Nigeria. The public should be educated on HEV, its modes of transmission, and intervention strategies including vaccination in accordance with the WHO recommendations for safe vaccine [57].

## Figures and Tables

**Table 1:** Characteristics and HEV IgM prevalence among study participants.

Variable	Ibadan pregnant women		Ibadan general population		Anambra general population	
	Frequency	Percentage (%)	Frequency	Percentage (%)	Frequency	Percentage (%)
Age						
20 and less	5	2.0	28	11.2	41	16.4
21–30	112	44.8	81	32.4	64	25.6
31–40	125	50.0	74	29.6	51	20.4
41–50	8	3.2	37	14.8	47	18.8
51 and above	-	-	30	12.0	47	18.8
Sex						
Male	-	-	73	29.2	113	45.2
Female	250	100	177	70.8	137	46.8
Total	250	100	250	100	250	100
HEV IgM						
Positive	1	0.4	0	0	0	0
Negative	249	99.6	250	100	250	100
Gestation age						
1st trimester	44	17.6	-	-	-	-
2nd trimester	132	52.8	-	-	-	-
3rd trimester	74	29.6	-	-	-	-

**Table 2:** HEV acute infection among the pregnant women.

Age	1st trimester			2nd trimester			3rd trimester		
	Not tested	Not positive	Not negative (%)	Not tested	Not positive	Not negative (%)	Not tested	Not positive (%)	Not negative (%)
20 and less	1	0	1 (100)	3	0	3 (100)	1	0	1 (100)
21–30	18	0	18 (100)	53	0	53 (100)	41	0	41 (100)
31–40	22	0	22 (100)	71	0	71 (100)	31	1 (3.2)	30 (96.8)
41–50	3	0	3 (100)	5	0	5 (100)	0	0	0
